# Inferior alveolar nerve injury with laryngeal mask airway: a case report

**DOI:** 10.1186/1752-1947-5-122

**Published:** 2011-03-29

**Authors:** Deepak Hanumanthaiah, Sarmad Masud, Anil Ranganath

**Affiliations:** 1 Department of Anaesthesia, Cork University Hospital, Cork, Ireland; 2 Department of Anaesthesia, Our Lady of Lourdes Hospital, Drogheda, Ireland

## Abstract

**Introduction:**

The incidence of damage to the individual cranial nerves and their branches associated with laryngeal mask airway use is low; there have been case reports of damage to the lingual nerve, hypoglossal nerve and recurrent laryngeal nerve. To the best of our knowledge we present the first reported case of inferior alveolar nerve injury associated with laryngeal mask airway use.

**Case presentation:**

A 35-year-old Caucasian man presented to our facility for elective anterior cruciate ligament repair. He had no background history of any significant medical problems. He opted for general anesthesia over a regional technique. He was induced with fentanyl and propofol and a size 4 laryngeal mask airway was inserted without any problems. His head was in a neutral position during the surgery. After surgery in the recovery room, he complained of numbness in his lower lip. He also developed extensive scabbing of the lower lip on the second day after surgery. The numbness and scabbing started improving after a week, with complete recovery after two weeks.

**Conclusion:**

We report the first case of vascular occlusion and injury to the inferior alveolar nerve, causing scabbing and numbness of the lower lip, resulting from laryngeal mask airway use. This is an original case report mostly of interest for anesthetists who use the laryngeal mask airway in day-to-day practice. Excessive inflation of the laryngeal mask airway cuff could have led to this complication. Despite the low incidence of cranial nerve injury associated with the use of the laryngeal mask airway, vigilant adherence to evidence-based medicine techniques and recommendations from the manufacturer's instructions can prevent such complications.

## Introduction

The incidence of damage to the individual cranial nerves and their branches associated with laryngeal mask airway (LMA) use is low; there have been case reports of damage to the lingual nerve [1], hypoglossal nerve [2] and recurrent laryngeal nerve [3]. Occlusion of the pharyngeal mucosal perfusion and direct compression of the surrounding structures by the LMA cuff or tubing can lead to pharyngolaryngeal morbidity [4-6]. High cuff volume and/or pressure and suboptimal use of the LMA are the most important etiological factors associated with these complications. We report an unusual case of vascular compression leading to inferior alveolar nerve injury, associated with LMA use, which lasted for two weeks.

## Case presentation

A 35-year-old man, height 175 cm, weight 85 kg and American Society of Anesthesiologists (ASA) grade I, underwent elective repair of his anterior cruciate ligament. He was a Caucasian man. He had no significant medical history. On examination, his airway was Malampatti grade I with no anticipated difficult intubation. Anesthesia was induced with propofol 2.5 mg/kg and supplemented with fentanyl 1 μg/kg. Face mask ventilation was easy. A disposable (Portex soft seal) LMA size 4 was inserted by an experienced user after lubrication with a water-based jelly. The cuff was inflated with 30 mL of air and secured in position with adhesive tape. Our patient's head was placed in a neutral position and initially there were movements of the head and neck while obtaining optimal positioning for the procedure. Our patient remained in the supine position throughout his surgery. Anesthesia was maintained with sevoflurane and nitrous oxide of 66% in oxygen with spontaneous ventilation. The LMA remained *in situ *for 120 minutes. There were no adverse events during maintenance or emergence from anesthesia. The LMA was removed with the cuff deflated when our patient responded to verbal commands. There was no blood on the LMA at removal.

Our patient noticed numbness in his lower lip in the recovery room. Sensory loss was confirmed by examination. There were no signs of oral trauma. Our patient also developed extensive scabbing of the lower lip on the second day after surgery. The numbness and scabbing started improving after a week, with complete recovery after two weeks.

## Discussion

LMA is one of the most widely used airway devices globally. Despite the non-invasive nature of the device, pharyngolaryngeal morbidity has been associated with it [4-6]. Most of these complications result from direct or indirect compression of the neurovascular structures. These include sore throat, damage to lingual, recurrent laryngeal, hypoglossal nerves, vocal cord paralysis, alteration of taste and speech, tongue cyanosis and swelling. The pressure exerted by the LMA cuff or its tubing and suboptimal use of the LMA are the most important predisposing factors. Other rare factors include lateral positioning of the patient [7], lidocaine lubricant [8], staff inexperience [9] and alternative insertion techniques [10].

In the case of our patient, we believe that the injury to the inferior alveolar nerve resulted from vascular compression. The inferior alveolar nerve is the largest branch of mandibular nerve and travels immediately posterior to the lingual nerve initially, and then along with the inferior alveolar vessels between the sphenomandibular ligament and the ramus of mandible. The nerve traverses the mandibular canal and divides into two branches. The inferior alveolar nerve lies superficially between the last molar tooth and the ramus of the mandible. An anesthetic block at this site leads to numbness of the lower lip. We believe that the compression occurred at this particular point. This also explains why the lingual nerve was spared.

In our review of the surgical procedure, multiple factors were identified that could have led to the injury. These were: use of maximum volume of air for cuff inflation, use of nitrous oxide, movement of our patient's head and neck during surgical positioning, inappropriate size of the LMA, unregulated cuff pressure and inappropriate securing method.

We inflated the cuff with 30 mL of air, which is the maximum volume for a size 4 LMA. The cuff should ideally be inflated to achieve an intra-cuff pressure of 60 cm H_2_O [11] or just seal pressure [12]. The inflation amounts mentioned for a given size of the LMA are the maximum inflation volumes. Frequently, only half the maximum volumes are sufficient [13]. Inflating the LMA with the maximum amount of air makes the cuff become more rigid and thus makes it less adaptable to the various contours of the pharynx. This can lead to pharyngeal mucosal ischemia and poor positioning, with the cuff sitting in the oral cavity. There is a possibility that direct compression of the LMA over the lower lip or indirect teeth bite could have led to this complication.

When nitrous oxide is used [14], it is always recommended that the cuff pressure should be periodically checked and gas intermittently withdrawn to maintain just 'seal pressure'. It has been shown that the pressure exerted by the cuff is not evenly distributed [15]. Moreover, the pressure exerted on the pharynx by the non-inflatable portions of the LMA, that is, the back plate and tubing, cannot be measured and are hence often overlooked.

The selection of size and the securing method of the LMA might have been a considerable factor as well. We used a size 4 LMA with our patient. There is evidence from randomized controlled studies and recommendations in the manufacturer's instruction manual to use a size 5 LMA in men and size 4 or 5 in women. If the size of the LMA is too small, there is an increased chance of poor positioning and overinflation of the cuff in an attempt to attain the maximum seal. However, there is a widespread tendency for anesthetists to use a size 4 for men and size 3 for women [15]. We did not follow the procedure as given by the manual while securing the LMA. This could also have led to displacement of the LMA.

Cranial nerve injuries associated with the use of the LMA are well established but rare complications. These usually present within 48 hours of surgery and resolve spontaneously over a period of weeks or months. Unless there is a strong suspicion of the cause, they might go unnoticed and be attributed to other causes.

## Conclusion

We report the first case of vascular occlusion and injury to the inferior alveolar nerve, causing scabbing and numbness of the lower lip. This is an original case report mostly of interest to anesthetists who use the LMA in day-to-day practice. Excessive inflation of the LMA cuff could have led to this complication. Despite the low incidence of cranial nerve injury associated with use of the LMA, vigilant adherence to evidence-based medicine techniques and recommendations from the manufacturer's instruction manual can help prevent such complications.

## Consent

Written informed consent was obtained from the patient for publication of this case report and any accompanying images. A copy of the written consent is available for review by the Editor-in-Chief of this journal.

## Competing interests

The authors declare that they have no competing interests.

## Authors' contributions

DH was responsible for writing the manuscript and for collecting and analyzing the references. SM was responsible for the follow-up of the case and was also a major contributor to writing the manuscript. AR was responsible for proofreading the manuscript and for revising it. All authors have read and approved the final manuscript.
