# Inferior alveolar nerve injury with laryngeal mask airway: a case report

**DOI:** 10.1186/1752-1947-5-560

**Published:** 2011-11-30

**Authors:** Deepak Hanumanthaiah, Anil Ranganath

**Affiliations:** 1 Department of Anaesthesia, Cork University Hospital, Cork, Ireland; 2 Department of Anaesthesia, Our Lady of Lourdes Hospital, Drogheda, Ireland

## Correction

Following the publication of our article [1] it was brought to our attention that we inadvertently used the registered trademark of the Laryngeal Mask Company Limited (LMA) as the abbreviation for laryngeal mask airway. A Portex^® ^Soft Seal^® ^Laryngeal Mask was used and not a device manufactured by the Laryngeal Mask Company.

All abbreviations for laryngeal mask airway written as LMA should be written in full as laryngeal mask airway.
